# New-onset autoimmune thyroid disease following COVID-19

**DOI:** 10.5415/apallergy.0000000000000145

**Published:** 2024-05-30

**Authors:** Öner Özdemir

**Affiliations:** 1 Division of Allergy and Immunology, Department of Pediatrics, Research and Training Hospital of Sakarya University Medical Faculty, Sakarya, Türkiye

## To the editor,

I have read the article titled “Thyroid disease post-COVID-19 infection: Report of a case with new-onset autoimmune thyroid disease” by Trinh et al. [1] with great interest. While reading this interesting rare case, I was puzzled by a few points that I could not comprehend.

First; in the article, the discrepancy between the time and dates of the onset of hyperthyroidism post-COVID-19 in the abstract, introduction, and discussion is very striking. In a few places, it is said to be 4 weeks (the first paragraph of the introduction and conclusion), and 6 weeks in the first paragraph under the case report, but when the data are calculated from the dates, it is 5 weeks (February 14, 2022–March 21, 2022), but in the abstract it is 8 weeks later, which is even more confusing.

Second; in this case report, standard abbreviations were not used when describing the patient and writing laboratory data. The units (U/L) versus (mIU/L) and abbreviations (TGAb) of laboratory values appear different in the text and table, which is perplexing [1].

Also, COVID-19 is the name of the disease caused by SARS-CoV-2 infection. Therefore, we believe that “post-COVID-19 infection or pre-COVID-19 infection” definitions and terms are not correct in the article text and the title [1].

Third; even in an article from 2021 literature [2], 5 cases of SARS-CoV-2 infection-induced Graves’ disease (autoimmune hyperthyroidism) were already mentioned by 3 independent groups 2 from Spain and one from the United States [2-5]. While it was surprising for us that the authors here mentioned 3 cases in this article published in March 2023 [1]. In this article, the references between 3 and 5 that we have cited here are probably unavailable or may have been overlooked. Our references between 3 and 5 belong to the years 2020 and 2021 are not given in the references of this article [1].

I would like to thank the authors for this nice case report raising awareness of SARS-CoV-2 infection-induced hyperthyroidism. This is a case report that later paved the way for future research on SARS-CoV-2 infection-induced autoimmune hyperthyroidism as well.

## Acknowledgment

None.

## Conflicts of interest

The authors declare no conflicts of interest.

## Author contribution

Öner Özdemir has done everything.
